# Tell Us What You Really Think: A Think Aloud Protocol Analysis of the Verbal Cognitive Reflection Test

**DOI:** 10.3390/jintelligence11040076

**Published:** 2023-04-21

**Authors:** Nick Byrd, Brianna Joseph, Gabriela Gongora, Miroslav Sirota

**Affiliations:** 1 Intelligence Community Postdoctoral Research Fellowship Program, Stevens Institute of Technology, Hoboken, NJ 07030, USA; 2 AIOps, IBM, Atlanta, GA 30319, USA; 3 College of Business, Florida State University, Tallahassee, FL 32306, USA; 4 Department of Psychology, University of Essex, Colchester CO4 3SQ, UK; msirota@essex.ac.uk

**Keywords:** cognitive reflection test, think-aloud protocol analysis, psychometrics, judgment- and decision-making, heuristics and biases

## Abstract

The standard interpretation of cognitive reflection tests assumes that correct responses are reflective and lured responses are unreflective. However, prior process-tracing of *mathematical* reflection tests has cast doubt on this interpretation. In two studies (N = 201), we deployed a validated think-aloud protocol in-person and online to test how this assumption is satisfied by the new, validated, less familiar, and non-mathematical verbal Cognitive Reflection Test (vCRT). Verbalized thoughts in both studies revealed that most (but not all) correct responses involved reflection and that most (but not all) lured responses lacked reflection. The think-aloud protocols seemed to reflect business-as-usual performance: thinking aloud did not disrupt test performance compared to a control group. These data suggest that the vCRT usually satisfies the standard interpretation of the reflection tests (albeit not without exceptions) and that the vCRT can be a good measure of the construct theorized by the two-factor explication of ‘reflection’ (as deliberate and conscious).

## 1. Introduction

If you were running a race and you passed the person in second place, what place would you be in now? The standard interpretation of a problem like this assumes that the answer that comes quickly and effortlessly to many people’s mind is “first place”. However, upon reflection, many people realize that the correct answer is “second place”. This problem is considered a test of reflection because it is designed to lure us toward a particular response that, upon reflection, we can realize is incorrect (Byrd 2022b, 2022c). Thus, the standard interpretation of reflection labels lured responses with ‘unreflective’ and correct responses with ‘reflective’ (Pennycook et al. 2015a).

Since the introduction of the Cognitive Reflection Test (Kahneman and Frederick 2002), theories of reflection have advanced (Evans and Stanovich 2013). In the midst of this progress, some theorists distilled dozens of reflective-unreflective distinctions (see Frankish 2010, Table 1) down to just two somewhat orthogonal distinctions: automatic versus deliberate processing and conscious versus unconscious representations (Shea and Frith 2016). According to this two-factor explication of ‘reflection’, *reflective* thinking involves more consciousness representation (or awareness) of the relevant reasoning and more deliberate inhibition (as opposed to immediate acceptance of one’s initial impulse) and *unreflective* thinking is less consciously represented and more automatic (Byrd 2022c). In other words, the *contents* of reflective reasoning are more accessible (e.g., to explain verbally) and the *process* of reflective thinking involves more reconsideration (e.g., doubting or double checking) than unreflective thinking (Byrd 2019). Theorists have also posited that the need for reflection may depend on context: in familiar reasoning domains, unreflective reasoning may be able to achieve desirable results, but in unfamiliar domains overcoming mistakes or biases might require some reflection (Pennycook et al. 2015a).

As theories of reflection progressed, so did our understanding of reflection tests. Some researchers have challenged the standard interpretation of mathematical reflection tests (mCRTs for short) and the two-factor explication of ‘reflection’ (Stanovich 2018). Indeed, some have found that most (67%) of those who answered a reflection test correctly after deliberation had already answered it correctly under time pressure or cognitive load before deliberation (Bago and De Neys 2019). While that two-response paradigm has helped test the default interventionist account of reflection—which posits that correct responses involve intervening on a default (a.k.a., lured) response (Evans 2007)—the paradigm overlooks plenty of useful information about the process of solving reflection test problems. So, others have listened to every word test-takers utter while thinking aloud during the reflection test. One such think-aloud study also “raise[d] doubts” about the standard interpretation of mCRTs: *most* (77%) correct responses on the mCRT “started … with the correct answer” and *many* (39%) lured responses on the mCRT involved “reflect[ing] on the… first response” (Szaszi et al. 2017). Some psychometric investigations of reflection tests suggest that these correct-but-unreflective and lured-yet-reflective responses might be explained by domain familiarity (Purcell et al. 2021), intelligence (Thompson and Johnson 2014), or strategy (Markovits et al. 2021). Although the predictive value of the mCRT remains after retaking the test (Białek and Pennycook 2018; Stagnaro et al. 2018), the best predictor of mCRT performance is often general math test performance (Attali and Bar-Hillel 2020; Erceg et al. 2020). So mCRTs may track not only reflection, but also mathematical competence (a.k.a., numeracy) and other factors.

### 1.1. The Verbal Cognitive Reflection Test

Sirota and colleagues (2021) developed and validated a new, 10-item, *non-mathematical* variant of Shane Frederick and colleagues’ (Frederick 2005; Kahneman and Frederick 2002) well-known (mathematical) cognitive reflection test (mCRT) to address familiarity and numeracy problems (Byrd 2022d). One item from Sirota and colleagues’ verbal cognitive reflection test (or vCRT for short) is the opening example: “If you were running a race, and you passed the person in 2nd place, what place would you be in now?” Multiple studies found that the vCRT enjoys high internal consistency, high test-retest reliability, and less association with general mathematical ability than the mCRT—even when translated to other languages and contexts (Sobkow et al. 2023). This suggests that the vCRT is a promising supplement or replacement for the mCRT in many research contexts.

### 1.2. Think-Aloud Protocol Analysis

Researchers have long called for investigation into the content and process of reflection rather than just the outcome (Stromer-Galley 2007). Fortunately, Ericsson and colleagues have developed and validated concurrent think-aloud protocols (Ericsson 2003; Ericsson and Simon 1993) that have been shown to overcome some well-known problems of early verbal report protocols such as confabulation (e.g., Wilson and Nisbett 1978) or performance interference on problems that seem unsolvable without some sort of insight (e.g., Schooler et al. 1993). For example, asking participants to *verbalize* or *recall* (rather than explain or justify) their thinking does not necessarily impair task performance or produce verbal reports inconsistent with their observed performance (Fox et al. 2011; Petitmengin et al. 2013). Concurrent verbalization can even *help* people think of a word that forms common compound words with three other words—e.g., thinking ‘tree’ when presented with ‘apple’, ‘family’, and ‘house’ (Ball and Stevens 2009; see also Blech et al. 2020). So, asking participants to think aloud while solving a problem (i.e., concurrently) may help participants tell us what they were really thinking better than retrospective requests to “explain”, which can imply that participants should verbalize a justification (rather than a recollection) of what they were thinking (Ericsson and Simon 1980). Evans and colleagues fruitfully applied this realization about concurrent verbal reports to logical reflection tests to arbitrate between “rationalist” and “two-factor” or “dual process” theories of reasoning (Evans et al. 1983). Szaszi and colleagues also used such think-aloud methods to investigate the cognitive processes involved in solving the original CRT (2017) (Szaszi et al. 2017). So, concurrent think-aloud protocols may also be useful for tracing vCRT test-takers’ thought processes and determining whether thinking aloud has a “reactive” effect on test performance (Ericsson 2003).

### 1.3. The Current Research

Our primary goals were (a) to test whether thinking aloud changed reflection test performance, (b) beta test an online think-aloud platform, (c) quantify the deviation between the standard interpretation of reflection tests and the two-factor explication of reflection, (d) to assess how vCRT performance depends on vCRT familiarity, and (e) assess the default interventionist account of reflection test responses. We pre-registered two hypotheses. First, thinking aloud during the vCRT will provide evidence of correct-but-unreflective responses and lured-yet-reflective responses. Second, thinking aloud will not significantly hinder vCRT performance—i.e., it will either not impact or improve reflection test performance. The results of an experiment and a follow-up study produced the hypothesized outcomes. They also detected that two-factor explication of ‘reflection’ strongly, albeit imperfectly, correlated with the standard interpretation of reflection test performance. All manipulations, measures, and exclusions are reported. All APA and IRB ethical guidelines were followed. Pre-registered hypotheses, methods, analytic strategy, data, and R scripts are on the Open Science Framework: https://osf.io/rk3jq.

## 2. Study 1

The first study primarily aimed to test the effect of thinking aloud on final responses to vCRT questions. The secondary aims were to test the difference in vCRT performance between familiar and naïve participants, the correlations between the standard interpretation of reflection tests and more recent explications of ‘reflection’, as well as the rate of correct-but-unreflective and lured-yet-reflective responses.

### 2.1. Method

*Participants*. People were recruited from public spaces on a university campus in the Southeastern United States. We pre-registered a target sample size of 100 participants—50 participants per condition (Simmons et al. 2013). After months of recruitment, reaching the pre-registered sample size with the in-person protocol became ethically and practically untenable because the World Health Organization announced a global pandemic (Ghebreyesus 2020), the university campus closed, and the university IRB announced that all in-person data collection must cease until further notice (Office for Human Subjects Protection 2020). Since the protocol could not be replicated online, we had to halt data collection after recruiting only 99 participants (mean age = 23.41 years; 48 identified as women, 50 as men, and 1 did not select a gender; 51 identified as White, 15 as Black, 16 as Hispanic or Latino, 1 as Pacific Islander, and 16 as other ethnicity).

### 2.2. Procedure and Materials

*Manipulation*. After consenting to participate, participants navigated to a Qualtrics survey using a QR code where they were randomly assigned to either a think-aloud condition or a control condition. To ensure that participants in both conditions completed the survey in front of a researcher, they were asked to remain at the research table until the end of the survey to receive their compensation: entry to win the smart speaker, water bottle, or books that were on the table.

*Think-aloud protocol*. Participants randomly assigned to the think-aloud condition were prompted to request instructions from a researcher. After the researcher explained the think-aloud protocol to participants, participants had a chance to ask for clarification and consent by selecting a button labeled “I received and understand the instructions from the researcher”. Then a researcher began an audio recording on a smartphone and the participant practiced thinking aloud on a pre-survey task, “To practice thinking aloud, please say this sentence aloud, followed by the following number ….” The number each participant read aloud was generated randomly and used to anonymously pair survey responses with each corresponding think-aloud recording. Participants were reminded to think aloud as needed throughout the survey.

**Figure A1:**
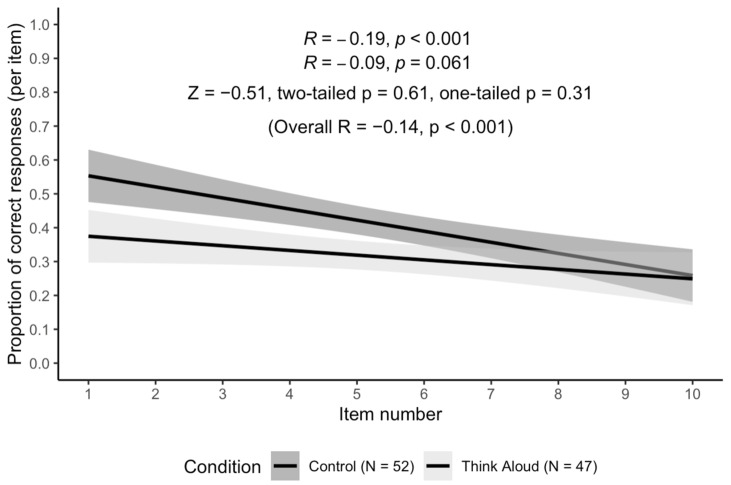
Practice effect tests in Study 1 (N = 99). Performance did not increase but decreased during the survey. The magnitude of this decrease was not dramatically different between conditions (Z-score). Error bands indicate standard error.

*Verbal Cognitive Reflection Test*. Participants completed the 10-item verbal Cognitive Reflection Test or vCRT (Sirota et al. 2021). Item order was fixed for all participants—no practice effects were observed (Appendix A Figure A1)—and responses were typed into text boxes. Following the standard interpretation of reflection tests, reflective scores were computed by summing correct responses (e.g., 2nd place) and unreflective scores were computed by summing lured responses (e.g., 1st place) on these verbal reasoning items. Non-lured incorrect responses were only 1.67% of all responses, lower than what has been observed for a widely used mCRT (Stupple et al. 2017, Table 5).

*Questions about lures*. To test whether correct responses followed lured responses—a la the default-interventionist account of reflection testing (Howarth and Handley 2016)—participants reported whether the lured response occurred to them after they submitted each reflection test answer. For instance, after answering the aforementioned question about passing the racer in 2nd place, participants were asked, “Have you thought at any point that ‘1st place’ could be the answer?”

*Deliberateness and consciousness in think-aloud recordings*. The two-factors explication of ‘reflection’ holds that reasoning is reflective when if it is more deliberate and more consciously represented (Shea and Frith 2016). Reasoning is said to be deliberate when it does not merely accept the initial, automatic response and is said to be conscious when participants can articulate parts of their reasoning (Byrd 2019, 2022c). So, two student raters (who were not aware of the project’s hypotheses), and then the first author, rated each response. Before raters became aware of participants’ final answers, raters rated each response’s deliberateness—i.e., whether the participant verbally reconsidered their initial response (Light’s *Κ*_avg_ = 0.59)—and conscious representation—i.e., whether the participant verbalized a reason for or against any response (Light’s *Κ*_avg_ = 0.43). Rating options included “yes”, “no”, and “indeterminate”, and the “moderate” agreement between raters allowed each participant’s “yes” ratings to be averaged (Landis and Koch 1977, p. 165).

*Test familiarity*. Prior work found that many participants were already familiar with reflection test questions and that such familiarity may be the best predictor of reflection test performance (Byrd 2022d; Stieger and Reips 2016). So the raters also rated whether each participant mentioned being familiar with any of the vCRT items (Light’s *Κ* = 0.82). Determinations were labeled “yes”, “no”, and “indeterminate”. The familiarity parameter was the average number of “yes” ratings per participant.

### 2.3. Results

We tested the rate of correct-but-unreflective responses and lured-yet-reflective responses on the vCRT, the effect of thinking aloud on reflection test performance, the correlation between standard reflection test scoring and recent explications of ‘reflection’, and the correlation between test familiarity and test performance.

*Correct-but-unreflective and lured-yet-reflective responses*. Think aloud verbal reports sometimes deviate from the standard interpretation of reflection tests. We expected some people to arrive at correct answers prior to reflection and without first thinking of the lured answer, and to be lured into accepting particular incorrect answers despite sustained reflection. Table 1 confirms this pre-registered expectation: the standard interpretation of correct and lured responses usually but imperfectly agrees with the two-factor explication of ‘reflection’.

**Figure 1:**
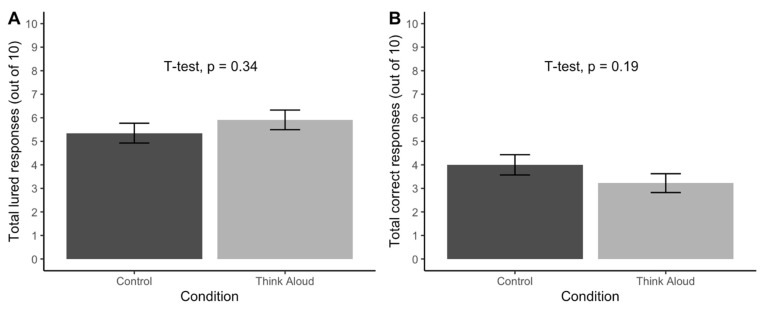
The effect of thinking aloud on (**A**) the number of lured responses and (**B**) the number of correct responses on the verbal cognitive reflection test (vCRT) in Study 1 (N = 99). Error bars represent a standard error.

*Thinking aloud did not impact performance*. Figure 1 affirms our pre-registered hypothesis and prior meta-analytic work (Fox et al. 2011): we did not detect an interference effect of thinking aloud on the number of lured or correct responses on the vCRT.

**Figure 2:**
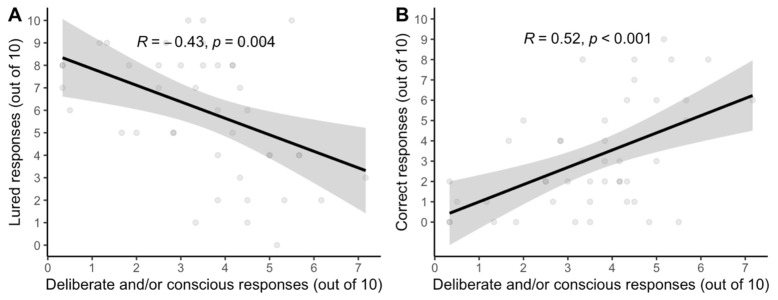
Correlations between the “two-factor” coding of reflective responses (deliberate and/or conscious) and (**A**) the number of lured—or so-called “unreflective”—responses and (**B**) the number of correct—or so-called “reflective”—responses on the verbal reflection test (vCRT) in the think-aloud condition of Study 1 (N = 47) with gray standard error bands.

*Two factor interpretation predicts the standard interpretation*. Regression analysis was employed to understand how well the standard interpretation of reflection tests aligns with dual process theorists’ two-factor explication of ‘reflection’. Figure 2 shows that they align well: the more that participants’ verbalizations involved deliberate reconsideration of *any* response or conscious articulation of reasons for *any* response, the less likely participants were to accept lured responses and the more likely participants were to *accept* correct responses. In other words, the degree to which participants’ *thinking* exhibited the two theoretical factors of reflection corresponded strongly with the degree to which participants’ *final answers* were deemed reflective or unreflective by the standard interpretation of test responses.

**Figure 3:**
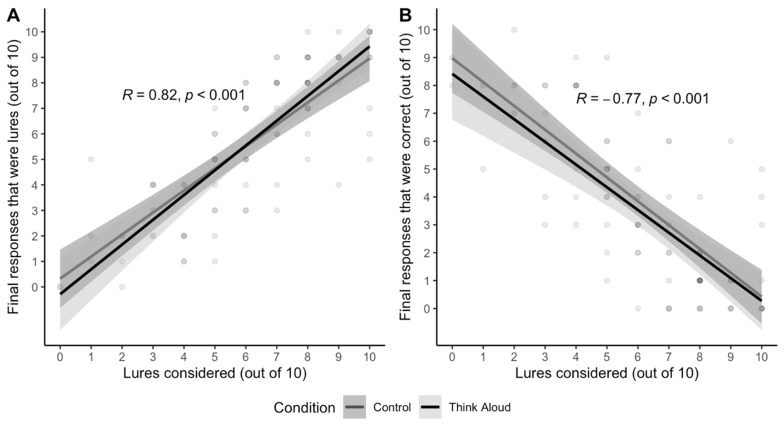
Correlations between *consideration* of lures and (**A**) *acceptance* of lures and (**B**) acceptance of correct responses on the verbal reflection test (vCRT) in Study 1 (N = 99). Gray bands represent a standard error.

*Consideration of lured responses*. Frederick (2005) observed that correct mCRT responses often involved consideration of the lured response. Some hypothesize that lures are appealing because they are more likely to feel correct (Thompson et al. 2013). If that is right, then people should not only be likely to *consider* lured responses, but those who consider lures should also be very likely to *accept* lures as their final answer. Figure 3 confirms this: consideration of lured responses *at some point* in each decision was relatively high (mean = 6.13, range = 0–10, S.D. = 2.59), and merely *considering* a lure almost perfectly predicted whether one *accepted* the lure as one’s final answer.

**Figure 4:**
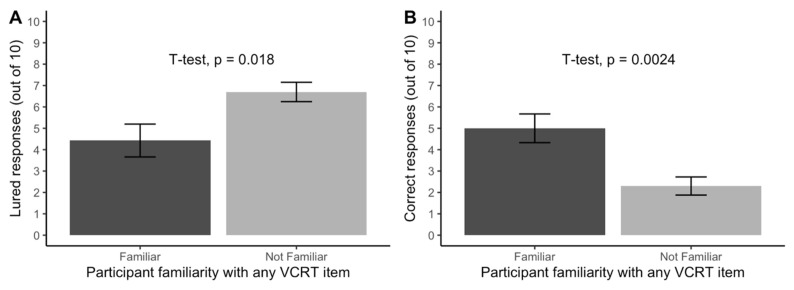
The number of (**A**) lured responses and (**B**) correct responses on the verbal reflection test (vCRT) among participants in the think-aloud condition of Study 1 (N = 47) depending on their unsolicited self-report of familiarity with the vCRT with standard error bars.

*Test familiarity predicted test performance*. In about 27% of think-aloud recordings, participants mentioned prior familiarity with at least one item on the vCRT—e.g., “I’ve seen these questions on TikTok”. Figure 4 shows a large difference in vCRT performance between familiar and naïve participants on both lured responses (*d* = −0.87) and correct responses (*d* = 1.13).

### 2.4. Discussion

These data suggest significant alignment between the dual process theorists’ two-factor explication of ‘reflection’ and the standard interpretation of reflection test answers. They also suggest that the university participants were largely naïve to the vCRT even though self-reported familiarity remained a strong predictor of the standard interpretation of vCRT performance.

One might wonder whether these results will replicate in a larger think-aloud validation of the vCRT. Fortunately, our initial results suggest that the think-aloud protocol will not significantly influence vCRT performance. So, a larger replication is methodologically possible. Unfortunately, large-scale, in-person think-aloud protocols are prohibitively time-consuming, tedious, or—during a pandemic—unethical. To overcome these challenges, we partnered with a startup to develop a platform for large-scale, online, think-aloud surveys.

## 3. Study 2

Study 2 aimed to replicate the findings of Study 1 in a new sample of participants and test the feasibility of online think-aloud survey methodology. To do this, we reproduced all of the instructions and measures in the think-aloud condition of Study 1 in an online audio survey platform, Phonic (Perrodin and Todd 2021; Phonic Inc. 2020).

### 3.1. Method

*Participants*. English-speaking monolingual participants were recruited from Prolific (Palan and Schitter 2018; Peer et al. 2017) for an expected $9.85/hour based on average completion time of the think-aloud condition of Study 1. To ensure data quality, Prolific alerted candidate participants that compensation would depend on their consent and ability to provide usable recordings of their thoughts throughout the survey. We aimed to double the pre-registered sample size of the think-aloud condition of Study 1 (N = 47), recruiting 102 participants (mean age = 30.38; 57 identified as women, 38 as men, and 7 did not select a gender; 85 identified as White, 3 as Black, 3 as Hispanic or Latino, and 11 as other ethnicity).

### 3.2. Procedure and Materials

*Phonic audio survey platform*. We used an online audio survey platform (Phonic) to record concurrent verbalizations; this service was provided in exchange for beta testing the new survey platform.

**Figure A2:**
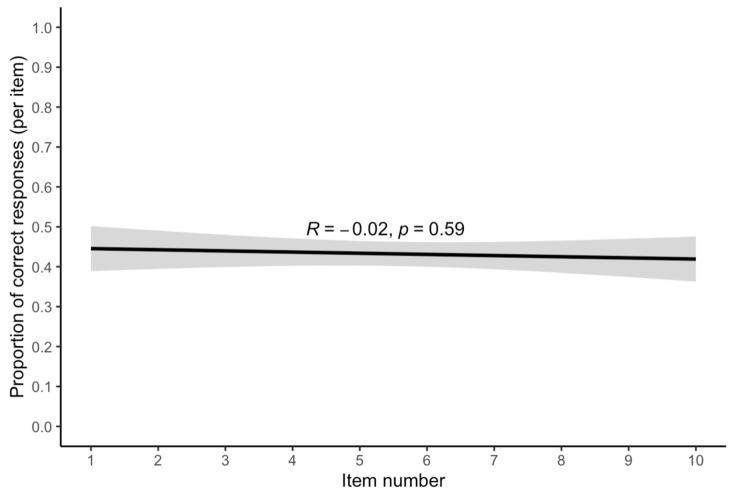
Practice effect test in Study 2 (N = 102). Performance did not increase or decrease during the survey. Error bands indicate standard error.

*Materials from Study 1*. All materials from the think-aloud condition of Study 1 were included in Study 2. Participants practiced thinking aloud before the survey and then thought aloud while completing the same 10-item vCRT with reminders to verbalize all their thoughts throughout the test—again, no practice effect was observed (Appendix A Figure A2). After each answer was submitted, participants answered the same follow-up question about whether they considered the lured response. The deliberateness, consciousness, and test familiarity of participants’ think-aloud recording for each question were rated by the principal investigator using the same rating options as Study 1. Replicating a result from Study 1, the rate of non-lured incorrect responses was very low (1.72%).

### 3.3. Results

We tested the correlation between test familiarity and test performance, the correlation between standard reflection test scoring and recent explications of ‘reflection’, as well as the rate of correct-but-unreflective responses and lured-yet-reflective responses on the vCRT.

*Correct-but-unreflective and lured-yet-reflective responses*. To test the agreement between the standard interpretation of reflection tests and more recent two-factor explications of ‘reflection’, the rates of correct-but-unreflective and lured-yet-reflective responses were determined by Prolific participants’ think-aloud recordings. Table 2 shows a replication of the preponderant yet imperfect agreement between the standard interpretation and the two-factor explication of reflection test answers.

**Figure 5:**
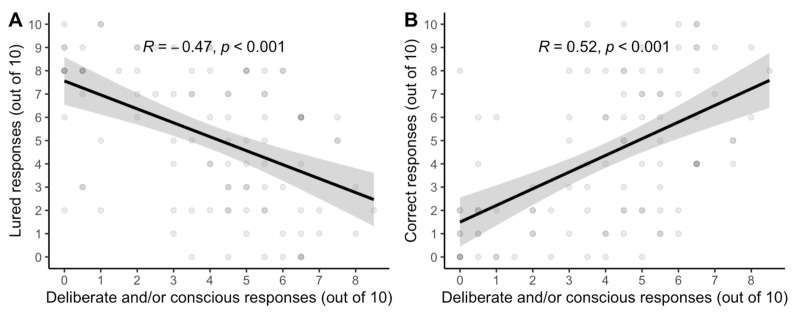
Correlations between the “two-factor” coding of reflective responses (deliberate and/or conscious) and (**A**) the number of lured—or so-called “unreflective”—responses and (**B**) the number of correct—or so-called “reflective”—responses on the verbal reflection test (vCRT) performance in Study 2 (N = 102). Gray bands represent a standard error.

*Two-factor interpretation predicts the standard interpretation*. Another regression analysis was employed to test how the standard interpretation of reflection tests aligns with dual process theorists’ two-factor explication of ‘reflection’. Figure 5 shows a replication of their correlation: increases in the number of participants’ responses that involved deliberate or conscious thinking—as determined by think-aloud recordings—correlated with significant decreases in the number of lured responses and significant increases in the number of correct responses.

**Figure 6:**
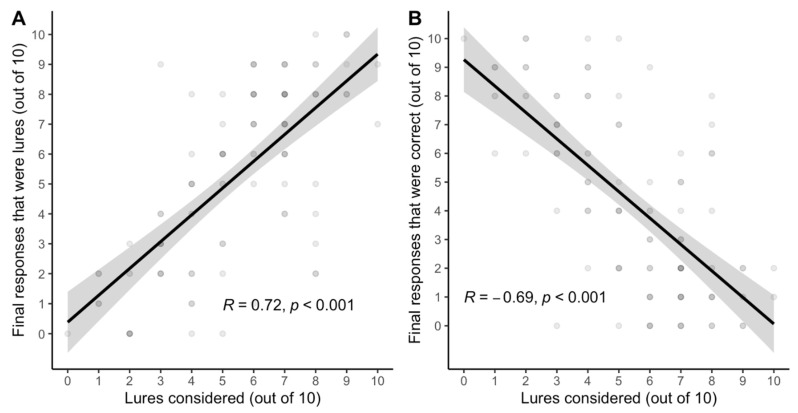
Correlations between consideration of lures and (**A**) *acceptance* of lures and (**B**) acceptance of correct responses on the verbal reflection test (vCRT) performance Study 2 (N = 102). Gray bands represent a standard error.

*Consideration of lured responses*. Figure 6 shows a replication of the strong feeling of rightness of lured responses. Indeed, lure *consideration* was not only relatively high (mean = 5.37, range = 0–10, S.D. = 2.38), it remained the best predictor of *accepting* both lured and correct responses on the vCRT.

**Figure 7:**
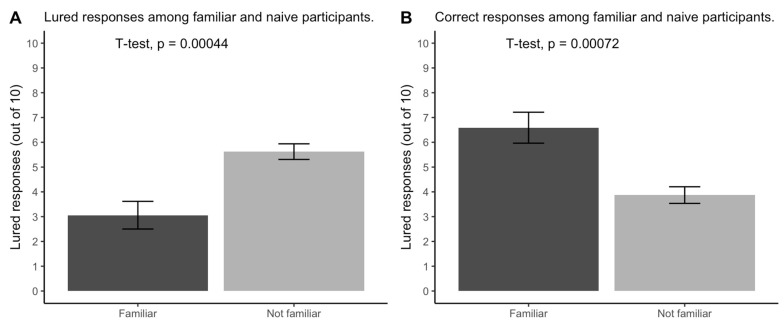
The number of (**A**) lured responses and (**B**) correct responses on the verbal reflection test (vCRT) among participants in Study 2 (N = 102) depending on their unsolicited self-report of familiarity with the vCRT. Error bars represent a standard error.

*Test familiarity predicted test performance*. In about 17% of think-aloud recordings, Prolific participants mentioned prior familiarity with at least one item on the vCRT—significantly less familiarity than the 27% familiarity among our university participants, *t* = −2.9, 95% CI [0.09, 0.24], *p* = 0.005. Figure 7 shows a replication of the large difference in vCRT performance between familiar and naïve participants for both lured responses (*d* = −0.91) and correct responses (*d* = 0.90).

## 4. General Discussion

In two think-aloud protocol studies, we found that our participants’ correct responses on the verbal cognitive reflection test occurred after thinking of the intuitively appealing but wrong initial responses (i.e., lures). When accepting lures as their *final* answers, our participants’ thinking typically lacked deliberative attempts to correct the initial, appealing, and wrong (lured) response. Exceptions to these two expected thinking trajectories on the vCRT comprised only 19–31% of responses—notably fewer than the 39–77% of exceptional responses detected for a widely used mCRT (Szaszi et al. 2017). Thus, our findings largely aligned with the standard interpretation of vCRT responses (Pennycook et al. 2015a) and more recent two-factor explications of reflective reasoning (Byrd 2019, 2022a, 2022c; Shea and Frith 2016). Nonetheless, the substantial minority of correct-but-unreflective and lured-yet-reflective responses indicate opportunities to reduce the measurement error of reflection tests (Machery 2021) to avoid falsely labeling some correct responses as reflective and some lured responses as unreflective or miserly (Toplak et al. 2014).

Further, we did not find that thinking aloud interfered with performance on the verbal reflection test. These studies also confirmed that most university participants and Prolific participants were naïve to the vCRT. Nonetheless, unsolicited think-aloud self-reports of familiarity with the vCRT were a strong predictor of the standard interpretation of vCRT performance in both studies. This evidence replicates and extends some of the promising features of the vCRT (Sirota et al. 2021).

### 4.1. Methodological Implications

The present studies also suggest that think-aloud protocols can reveal valuable and otherwise undetected nuance in cognitive reflection test performance. For instance, think-aloud recordings revealed that the standard interpretation of reflection test responses mislabeled 19–31% of responses as either reflective or unreflective.

This insight seems to increase the justificatory burden of employing the standard interpretation of reflection tests or of *not* employing the think-aloud protocols (Byrd 2022b) that more accurately detected the deliberate and conscious features of reflective thinking (Shea and Frith 2016). Even if researchers do not rethink their interpretation of or reliance on reflection tests, they may nonetheless need to justify the status quo.

In addition to implications for *measuring* reflection, there may also be implications for *manipulating* reflection. Recall the two-response paradigm of measuring reflection (e.g., Bago and De Neys 2019). It may not provide insight into the process that changes participants’ initial response, but it does indicate whether this initial response was already correct. So the two-response paradigm could be a compromise between the ease of the standard interpretation of reflection tests and the tedium of coding reflection test transcripts (e.g., Burič and Šrol 2020). Ideally, however, this second response would come after some sort of reflective task—as opposed to an imposed waiting period in which participants can do whatever they want—to ensure that observed changes are the result of reflection and not some other factor (Isler et al. 2020).

### 4.2. Theoretical Implications

Another result of the present studies was reliable support for the “feeling of rightness” explanation of reflection test performance (Pennycook et al. 2015b; Thompson et al. 2011). Most responses involved *consideration* of the lure and *consideration* of lures was the best predictor of *accepting* both lured and correct responses on the vCRT. If lures were not significantly more appealing than other possible responses, then it would be difficult to explain this preponderance of lure *consideration*, lure *acceptance*, and their strong correlation.

This may have implications for the debate between default interventionist accounts of reflection and their alternatives (Howarth and Handley 2016). Those who arrived at the correct response were very unlikely to have considered the lured response. In other words, the so-called reflective (i.e., correct) response on reflection tests may not usually involve intervening on a default (lured) response after all. Of course, these data confirm that there are *some* cases of reflective default intervention. So the current evidence may not falsify the default interventionist account so much as show that it is not an exhaustive explanation of reflection test performance.

### 4.3. Limitations

The current studies were limited by resources for listening to and coding think-aloud verbal reports. This resulted in minimal sample sizes for the research questions addressed in this paper (Simmons et al. 2013). Although the expected effects were detected—some in multiple populations, both in-person and online—there remains an opportunity for researchers with more resources to conduct larger-scale replications and extensions of the existing work. For example, researchers could annotate additional aspects of participants’ transcripts from reflection tests—e.g., whether overcoming faulty intuitions varies depending on the *kinds* of reasons participants consider: reasons for their initial response, reasons for subsequent responses, reasons against their initial response, or reasons against subsequent responses (Cullen et al. 2022). We look forward to seeing what other researchers discover with our and others’ (open) datasets. There are also opportunities for online think-aloud survey platforms to improve their speech transcription, sentiment analysis, and other features enough to automate and therefore scale up think-aloud protocol research. 

## 5. Conclusions

The present studies partially replicate and clarify existing validations of the verbal cognitive reflection test, thanks in part to novel online audio survey technology. Most participants are naïve to the test and the standard interpretation of reflection testing largely aligns with more advanced explications of reflective reasoning. Taken together with existing work showing that verbal reflection tests can have high internal consistency, high test-retest reliability, and less association with mathematical ability or gender, the present evidence suggests that the vCRT could be a promising supplement or replacement for widely used reflection tests. Nonetheless, there may still be opportunities to improve our understanding of reflection by redeploying online think-aloud protocols for larger-scale research. Thus, both verbal reflection tests and online think-aloud protocols are promising tools for advancing our understanding of reflective reasoning and its alternatives.

## Figures and Tables

**Table 1:** Standard and two-factor categorizations of reflection test responses based on think-aloud protocol analysis of responses to the verbal reflection test in Study 1. Example verbalization based on the following reflection test question: “If you were running a race, and you passed the person in 2nd place, what place would you be in now?”.

Category	Example Verbalization	Answer	Standard	Two-Factor	Rate
Correct-and-reflective	“1st obviously. No actually … 2nd.”	Correct	Reflective	Reflective	80.2%
Correct-but-unreflective	“2nd”	Correct	Reflective	Unreflective	19.8%
Lured-and-unreflective	“1st”	Lured	Unreflective	Unreflective	71.5%
Lured-yet-reflective	“I want to say 1st but, umm, yeah, 1st.”	Lured	Unreflective	Reflective	28.5%

**Table 2:** Standard and two-factor categorizations of reflection test responses based on think-aloud protocol analysis of responses to the verbal reflection test in Study 2. Example verbalization based on the following reflection test question: “If you were running a race, and you passed the person in 2nd place, what place would you be in now?”.

Categorization	Example Verbalization	Answer	Standard	Two-Factor	Rate
Correct-and-reflective	“1st. No … 2nd. Whoopsie”	Correct	Reflective	Reflective	68.5%
Correct-but-unreflective	“2nd”	Correct	Reflective	*Unreflective*	31.5%
Lured-and-unreflective	“1st”	Lured	Unreflective	Unreflective	75.8%
Lured-yet-reflective	“1st … or is that a trick? … I’d say 1st.”	Lured	Unreflective	*Reflective*	24.2%

## Data Availability

All data and analysis files available at https://osf.io/rk3jq/.

## Appendix A

*Think Aloud Instructions*

In this experiment we are interested in what you think about when you find answers to some questions that I am going to ask you to answer. In order to do this we ask you to THINK ALOUD as you work on the problem given.

What I mean by ‘think aloud’ is that I want you to tell me EVERYTHING you are thinking from the time you first see the question until you give an answer. I would like you to talk aloud CONSTANTLY from the time you start the survey to the time that you finish.

Please don’t plan out what you say or try to explain to me what you are saying. Just act as if you are alone in the room speaking to yourself.

It is most important that you keep talking. If you are silent for any long period of time I will ask you to talk.

Do you understand what we need you to do?

Good, now you can practice thinking aloud on this sample question.

[Ensure they read aloud (including the code) and continue to think aloud while practicing]

Ok. Now you will begin the survey.

[Use phrase like ‘remember to say your thoughts aloud’ if they are silent for a few seconds.]

[Thank them when they are done.]

**Modified Verbal Cognitive Reflection Test (Sirota et al. 2021)**

(1) Mary’s father has 5 daughters but no sons—Nana, Nene, Nini, Nono. What is the fifth daughter’s name probably?
  [correct answer: Mary, lured answer: Nunu]
  [Page break]
  “Do you remember thinking at any point that ‘Nunu’ could be the answer?”
  Yes No
(2) If you were running a race, and you passed the person in 2nd place, what place would you be in now?
  [correct answer: 2nd, lured answer: 1^st^]
  [Page break]
  “Do you remember thinking at any point that ‘1st’ could be the answer?”
  Yes No
(3) It’s a stormy night and a plane takes off from JFK airport in New York. The storm worsens, and the plane crashes-half lands in the United States, the other half lands in Canada. In which country do you bury the survivors?
  [correct answer: don’t bury survivors, lured answers: answers about burial location]
  [Page break]
  “Do you remember thinking at any point that survivor burial was an option?”
  Yes No
(4) A monkey, a squirrel, and a bird are racing to the top of a coconut tree. Who will get the banana first, the monkey, the squirrel, or the bird?
  [correct answer: no banana on coconut tree, lured answer: any of the animals]
  [Page break]
  “Do you remember thinking at any point that ‘bird’, ‘squirrel’, or ‘monkey’ could be the answer?”
  Yes No
(5) In a one-storey pink house, there was a pink person, a pink cat, a pink fish, a pink computer, a pink chair, a pink table, a pink telephone, a pink shower—everything was pink! What colour were the stairs probably?
  [correct answer: a one-storey house probably doesn’t have stairs, lured answer: pink]
  [Page break]
  “Do you remember thinking at any point that ‘pink’ could be the answer?”
  Yes No
(6) How many of each animal did Moses put on the ark?
  [correct answer: none; lured answer: two]
  [Page break]
  “Do you remember thinking at any point that ‘two’ could be the answer?”
  Yes No
(7) The wind blows west. An electric train runs east. In which cardinal direction does the smoke from the locomotive blow?
  [correct answer: no smoke from an electric train, lured answer: west]
  [Page break]
  “Do you remember thinking at any point the locomotive will produce smoke?”
  Yes No
(8) If you have only one match and you walk into a dark room where there is an oil lamp, a newspaper and wood—which thing would you light first?
  [correct answer: match, lured answer: oil lamp]
  [Page break]
  “Do you remember thinking at any point that ‘oil lamp’, ‘newspaper’, or ‘wood’ could be the answer?”
  Yes No
(9) Would it be ethical for a man to marry the sister of his widow?
  [correct answer: not possible, lured answer: yes, no]
  [Page break]
  “Do you remember thinking at any point that it is possible for a man to marry the sister of his widow?”
  Yes No
(10) Which sentence is correct: (a) “the yolk of the egg are white” or (b) “the yolk of the egg is white”?
  [correct answer: the yolk is yellow, lured answer: b]
  [Page break]
  “Do you remember thinking at any point that ‘a’ or ‘b’ could be the answer?”
  Yes No

*Scoring and Coding*

- Conditions: Control condition = 0. Think Aloud condition = 1.
- Standard coding: Sum correct and lured answers for reflective and unreflective parameters, respectively.
- From transcripts of think-aloud condition, create variables for (a) whether each participant reconsidered each their initial responses, (b) whether each participant verbalized any reason(s) for or against any response(s), and (c) whether a participant mentioned being familiar with any verbal reflection test question.
